# Calcidiol Status and Metabolic Syndrome in 40–60-Year-Old Women: Association with Metabolic Components

**DOI:** 10.3390/nu18142311

**Published:** 2026-07-14

**Authors:** Alexandra Tijerina-Sáenz, Ana Paula Romo-Uribe, Adbel Zaid Martínez-Báez, Elizabeth Solís-Pérez, Michel Stéphane Heya, Cristina Bouzas, Daniela Rodrigues, Josep A. Tur

**Affiliations:** 1 Facultad de Salud Pública y Nutrición, Universidad Autónoma de Nuevo León, Monterrey 64460, Mexico; 2 Research Group on Community Nutrition and Oxidative Stress, University of Balearic Islands–IUNICS, 07122 Palma de Mallorca, Spain; 3 Health Research Institute of the Balearic Islands (IdISBa), 07120 Palma de Mallorca, Spain; 4 CIBER Physiopathology of Obesity and Nutrition (CIBEROBN), Institute of Health Carlos III (ISCIII), 28029 Madrid, Spain; 5 Research Centre for Anthropology and Health (CIAS), Department of Life Sciences, University of Coimbra, 3000-456 Coimbra, Portugal; drdc@uc.pt

**Keywords:** vitamin D, calcidiol, metabolic syndrome, menopause, women, Mexico

## Abstract

Background: Metabolic syndrome (MetS) is a public health problem with high prevalence in adult Mexican women. Evidence suggest that vitamin D deficiency is prevalent among individuals’ metabolic disorders, obesity, and insulin resistance. Objective: To assess the association between calcidiol status (25D_3_) and metabolic syndrome in 40-to-60-year-old Mexican women. Methods: A retrospective cross-sectional study was conducted in 202 women from the northeast of Mexico. The diagnosis of MetS was established using the definition of the International Diabetes Federation. Calcidiol was quantified using a validated method of liquid chromatography (HPLC-UV). Results: The prevalence of MetS was 59.9% and calcidiol deficiency (<20 ng/dL) 67.8%. Women with MetS had lower calcidiol levels than those in the NoMetS group (9.0 ng/mL vs. 17.2 ng/mL; *p* = 0.003). Presence of MetS was associated with deficient calcidiol levels (*p* = 0.023). Most MetS components showed an increased likelihood, 1.7 to 9.1%, of the presence of MetS at deficient (<20 ng/dL) and low (<30 ng/mL) calcidiol levels. Conclusions: Maintaining calcidiol levels at or above 30 ng/mL has been stated as protective for 40–60-year-old women; however, vitamin D supplementation as a protective strategy against MetS cannot be recommended. Longitudinal and intervention studies are needed to clarify causality and evaluate the benefits of vitamin D in women with metabolic syndrome.

## 1. Introduction

Vitamin D is a fat-soluble vitamin that acts as a prohormone, and its most recognized function is the regulation of calcium and phosphorus metabolism to maintain bone homeostasis [1]. However, it was pointed out that Vitamin D participates in multiple metabolic and endocrine processes [2]. Vitamin D can be obtained through sun exposure, diet, and supplementation, although it is estimated that between 80% and 90% of this vitamin comes from cutaneous synthesis induced by ultraviolet radiation [3,4]. Foods with the highest vitamin D content include fatty fish, cod liver oil, and fortified foods [1]. Despite this, dietary intake of vitamin D has been reported to be insufficient in adult women, especially those in menopausal transition or postmenopause [5]. Storage and circulating levels of vitamin D are mainly determined and known as calcidiol, 25-hydroxyvitamin D_3_ or 25-hydroxycholecalciferol (25(OH)D_3_). Several international institutions such as the Endocrine Society, the International Osteoporosis Foundation (IOF) and the European Society for Clinical and Economic Aspects of Osteoporosis and Osteoarthritis (ESCEO) recommend a target level of 30 ng/mL (75 nmol/L) [6,7], mainly to maintain bone health. Postmenopausal women frequently exhibit low serum calcidiol levels, possibly related to reduced sun exposure, decreased physical activity, inadequate dietary intake, and vitamin sequestration in adipose tissue [8,9]. Hypovitaminosis D has been previously reported in 63% of Mexican adults [10] and 34.7% in Spanish adults [11] and associated with cardiovascular disease, diabetes mellitus, osteoporosis, and other metabolic disorders [12,13].

Menopausal transition typically occurs between the age of 40 and 56 years [14]. These stages are associated with significant hormonal and metabolic changes that contribute to increased abdominal adipose tissue, decreased lean mass, and alterations in glucose and lipid metabolism [15] that furthermore may develop to health complications such as metabolic syndrome (MetS). MetS is defined as a cluster of metabolic abnormalities that include abdominal obesity, hypertension, dyslipidemia, hyperglycemia, and insulin resistance, conditions that increase the risk of developing cardiovascular disease and type 2 diabetes mellitus [16]. In Mexico, MetS has a prevalence of 65.4% in adults aged 40–59-years-old, according to the 2018 National Health and Nutrition Survey (ENSANUT) [17], around 60% in postmenopausal women [18], and 57.2% in 40–60-year-old women [19]. Several factors, such as sedentary lifestyle, poor diet quality, increased energy intake, and decreased energy expenditure, contribute to the accumulation of visceral adipose tissue and the development of metabolic abnormalities associated with MetS [20,21].

Several observational studies suggest that calcidiol may play a protective role against metabolic syndrome due to its involvement in the regulation of insulin, inflammation, and the renin–angiotensin system [22]. However, the exact mechanisms explaining this relationship are not yet fully understood and research results remain controversial [9]. Some studies have found an association between low calcidiol status and MetS in adults [23], other studies with increased waist circumference, hyperglycemia, and insulin resistance [24,25], and others have not obtained a conclusive association [16]. Therefore, the aim of this study was to assess the association between calcidiol status and metabolic syndrome in 40-to-60-year-old Mexican women.

## 2. Methods

### 2.1. Design and Study Participants

A descriptive cross-sectional study was carried out in a convenience sample, *n* = 202, of 40–60-year-old women, residents of the metropolitan area of Monterrey, Nuevo Leon State, in the northeast of Mexico. According to the stages of reproductive aging workshop criteria, STRAW + 10, women were reproductive, with regular menstrual cycles or no present changes, and postmenopausal, with absence of menstrual cycle for ≥12 months [26].

The study was developed following the Declaration of Helsinki and approved by the Ethics Committee of the Faculty of Public Health and Nutrition of the Autonomous University of Nuevo León (Protocol ID: 21-FaSPyN-SA-19.TP). Written informed consent was obtained from each participant. Data collection was performed in the Centre for Research in Nutrition and Public Health of the Faculty of Public Health and Nutrition.

### 2.2. Metabolic Syndrome Definition

MetS was defined according to the IDF Worldwide Definition [27], abdominal obesity as waist circumference ≥ 80 cm, and two or more of the following components: triglyceride levels ≥ 150 mg/dL or antilipemic treatment, HDL-cholesterol < 50 mg/dL or specific treatment, blood pressure ≥ 130/85 mmHg or antihypertensive treatment, fasting glucose levels ≥ 100 mg/dL or antidiabetic treatment.

### 2.3. Anthropometrics and Body Composition

Waist circumference (WC) was measured to the nearest 0.1 cm using a non-stretch measuring tape (SECA 201, Hamburg, Germany) at the midpoint between the last rib and the iliac crest. Height was measured to the nearest millimeter using a digital stadiometer (SECA 274, Hamburg, Germany), with the participant’s head in the Frankfurt horizontal plane.

Bioelectrical impedance analysis (Inbody A120 & Software Lookin’Body 120, Inbody Co., Seoul, Republic of Korea) was used to measure the participant’s body weight (kg) to the nearest 100 g and to calculate the body mass index (BMI, kg/m^2^). BMI classification, as normal weight, overweight, and obese, was according to Norma Oficial Mexicana NOM-008-SSA3-2017 [28].

### 2.4. Blood Pressure Measurements

Systolic and diastolic blood pressure (SBP and DBP) was measured to the nearest 1 mmHg using a Beurer blood pressure monitor (BM19, Beurer Medical Instruments, Ulm, Germany) with participants seated in a resting chair using the left arm with the palm facing upward, according to Norma Oficial Mexicana NOM-030-SSA2-2017 [29].

### 2.5. Biochemical Assays

Venous blood samples were obtained from the antecubital vein in suitable vacutainers after 12 h overnight fasting, as established by the Proyecto de Norma Oficial Mexicana NOM-253-SSA1-2024 [30]. Blood samples were centrifuged at 3500 rpm for 12 min, then plasma was frozen at –80 °C until assays were performed. Biochemical assays were performed in the A25 automatic analyzer, software v4.1.1, and commercial assay kits were used to determine glucose (CV = 1.4%) at 505 nm, triglycerides (CV = 1.7%) at 505 nm, and HDL-cholesterol (CV = 2.9%) at 535 nm (BioSystems^®^ S.A, Barcelona, Spain).

### 2.6. Calcidiol (25D_3_) Status

Calcidiol (25D_3_) quantitation was done in plasma samples by an HPLC-UV method previously validated [31]. Briefly, the HPLC equipment had a UV-Vis diode-array detector and utilized an Acclaim^TM^ 120 C18 column (5 μm, 4.6 × 250 mm) with a flow rate of 1.2 mL/min of acetonitrile (ACN)–0.1% formic acid (2:1 *v*/*v*), a column temperature of 30 °C, and the standards and samples were maintained at 4 °C, with an injection volume of 100 μL. Detection of calcidiol was determined at 265 nm, with a retention time of 4.0 min. The method had a linearity of R^2^ = 0.9989, precision < 10%, recovery of 95% and detection and quantitation limits of 1.1703 and 3.5462 ng/mL, respectively. Plasmatic levels of calcidiol were deficient ≤ 20 ng/mL, insufficient 21–29 ng/mL, or sufficient ≥30 ng/mL [3]. The International Osteoporosis Foundation (IOF) position statement recommends levels of 30 ng/mL or above [6] for better health, especially in fracture prevention.

### 2.7. Vitamin D Intake

Information on vitamin D intake was obtained from the consumption of foods and beverages using a previously validated food frequency questionnaire (FFQ) [32], while the use of vitamin D supplements and their frequency of consumption was also detailed by participants. Dietetic and supplement information was analyzed in the software Food Processor^®^ version 15.0 (ESHA Research, Salem, OR, USA) and vitamin D intake was reported as international units per day (IU/day). Recommended vitamin D intake for Mexican women (51–70 years) is 10 μg/day or 400 IU/day [33].

### 2.8. Statistics

Data were analyzed for normality by the Kolmogorov–Smirnov test. Participants were stratified by the presence of metabolic syndrome: NoMetS vs. MetS. Numerical data are shown as median (quartiles) (Q1–Q3) and were calculated by the Mann–Whitney U test. Categorical data are shown as frequency (percentage), *n* (%), and differences between groups were analyzed by the Chi-squared test. Three groups of calcidiol levels were arranged as deficiency ≤20 ng/mL, insufficiency 21–29 ng/mL, and sufficiency ≥30 ng/mL. To determine the association between MetS components and calcidiol levels, frequency and percentage of participants at each level were analyzed using the Chi-squared test.

Logistic regression analysis was used to assess the association between the presence of MetS and its components according to three groups of calcidiol: at levels of deficiency ≤20 ng/mL, below 30 ng/mL (deficiency ≤20 ng/mL + insufficiency 21–29 ng/mL), and at sufficiency ≥30 ng/mL. Two models were calculated, the first model was adjusted for age, stage of aging, and BMI, while the second model was adjusted for age, stage of aging, BMI, vitamin D intake (foods + supplements), season at blood extraction and use of antilipemic treatment.

Analyses were performed in SPSS statistical software package (SPSS v.22 for Windows, IBM Software Group, Chicago, IL, USA) and significance was set at *p* < 0.05.

## 3. Results

Results on MetS components, calcidiol status and intake are shown in Table 1, grouped into NoMetS and MetS. The presence of metabolic syndrome was 59.9%. Age was similar between groups; overweight, 25.8 kg/m^2^, and obesity, 30.8 kg/m^2^, were present in the NoMetS and MetS groups, respectively. Significant differences were found between groups in all MetS components and vitamin D intake. WC was above 80 cm in both groups (85.5 cm in the NoMetS group vs. 97.0 cm in the MetS group). Median blood pressure in the MetS group, SBP at 123.0 mmHg and DBP at 79.0 mmHg, were higher than those levels in the NoMetS group. Serum glucose and triglyceride levels were lower in the NoMetS group; serum glucose and triglyceride levels were lower in the NoMetS group (*p* < 0.001), while HDL-cholesterol levels were low, below 50 mg/dL, in both groups, although slightly higher in NoMetS women. The median vitamin D intake in both groups was deficient, and even lower still in the MetS group, 198.9 IU/day in NoMetS vs. 120.3 IU/day in MetS. Only 23 women met the vitamin D intake requirement, 11 in the NoMetS vs. 12 in the MetS group. Along the study sample, eleven participants took vitamin D supplements. Furthermore, both groups showed deficient calcidiol levels but participants with MetS exhibited markedly lower levels than those NoMetS, 9.0 vs. 17.2 ng/mL (*p* < 0.001), respectively).

Frequency of participants showing MetS components and grouped according to plasma calcidiol levels as deficiency: <20 ng/mL, insufficiency: 21–29 ng/mL, and sufficiency: >30 ng/mL are shown in Table 2. The frequency of women with several MetS components (waist circumference, blood pressure, triglycerides, HDL-cholesterol, and glucose) were similar at different calcidiol levels. When considering the presence of MetS (yes), an association was observed with calcidiol deficiency (<20 ng/mL).

Most metabolic syndrome components (Table 3) were associated with the presence of MetS at calcidiol deficiency at ≤20 ng/dL, (OR: 1.017–1.083) and at below 30 ng/dL (OR: 1.018–1.090), HDL-cholesterol also demonstrated a negative association at both calcidiol levels (OR: 0.856–0.936). WC was not associated with MetS at deficiency levels, but at concentration below 30 ng/dL (OR: 1.090, *p* = 0.035), although the second model did not reach significance. At levels of sufficiency, ≥30 ng/dL, none of the MetS components showed any association with the presence of MetS in both proposed models.

## 4. Discussion

Current findings showed that 59.9% of the studied Mexican women have MetS, and 81.7% have low levels of calcidiol (25D_3_) in the form of deficiency (67.8%) or insufficiency (13.9%); whereas of the women who have MetS, 85.9% of them show low levels in the form of deficiency (75.2%) or insufficiency (10.7%). In the current study, the prevalence of calcidiol deficiency was about double of that in a study from Brazil, where of the 45–75-year-old women with MetS, 35.4% showed deficiency, and 32.6% insufficiency of calcidiol [34]. In a recent study in Croatia, calcidiol deficiency was at 55.1% in adult women (>28 years) [35] and there were similar proportions in a group of Colombian women [13].

One possible explanation for these findings may be that obtaining sufficient vitamin D from food is difficult, as its content varies from food to food [36]. There are few foods rich in this nutrient such as fatty fish, eggs, and liver [37,38]; however, frequent consumption of vitamin D-fortified foods, such as milk, fish, Manchego and Oaxaca cheeses, was associated with lower inflammation in adults with MetS [39]. In addition to differences in vitamin D intake among countries, methodological differences may also have contributed to the observed differences in calcidiol deficiency prevalence. Specifically, the use of different diagnostic criteria for MetS (IDF Worldwide Definition versus NCEP-ATP III) may have influenced the results. The IDF criteria use a lower waist circumference cutoff (>80 cm versus >88 cm), which tends to classify more women as having MetS [27]. Consequently, differences in the study population defined as having MetS could have affected the reported prevalence of calcidiol deficiency.

In addition, the variation in calcidiol levels among different countries may be due to other factors. Moreover, sun exposure has a high influence on vitamin D status [40], with 80 to 90% of vitamin D obtained through solar exposure, with less emphasis in obtaining it from the diet [3,4]. Accordingly, in this current study, a higher frequency of sufficient calcidiol levels was observed in blood samples taken in the summer (59.4%), followed by spring (17.8%), which are seasons with predominantly high sun exposure. Another possible explanation of low circulating calcidiol levels may be due to hepatic steatosis, which impairs hepatic enzyme metabolic pathways, and obesity-related chronic inflammation [16], mechanisms related to metabolic syndrome that may further reduce the expression and activity of the hepatic 25-hydroxylase CYP2R1 [41], thus reducing the conversion of vitamin D to calcidiol.

Moreover, an association between the presence of MetS and deficient calcidiol status has been found in this study [35]. Calcidiol deficiency may be due to low vitamin D intake as reported in both groups of women, 194.0 IU/day in NoMetS vs. 120.3 IU/day in MetS. Similarly, adults from Brazil with MetS did not meet the recommended daily intake of vitamin D, as reported intake (70.4 IU/day) was half of that reported in our current study; also no association between vitamin D intake and the presence of MetS was determined [42].

Calcidiol deficiency is hardly associated with MetS. Low levels of calcidiol are linked to increased insulin resistance, abdominal obesity, hypertension, and altered cholesterol and triglyceride levels. The relationship between the two factors is bidirectional and is based on the following mechanisms: Vitamin D acts as a hormone that regulates multiple metabolic processes; it improves the function of pancreatic beta cells, helping to lower glycemia; it reduces chronic inflammatory markers often associated with obesity; and it helps regulate the renin–angiotensin–aldosterone system, which is key to maintaining blood pressure at normal levels [16,43].

Large WC has been previously associated with deficient serum calcidiol levels [24,44,45]. One study in pre- and postmenopausal women reported that a WC > 88 cm increased the risk of calcidiol deficiency up to fifteenfold (*p* < 0.001) [46]. Another study reported significant differences in patients without plasma calcidiol deficiency with a smaller WC *(p* = 0.012) [16]. In the current study, the median WC was reported to be >85 cm in both groups, surpassing the IDF criteria of ≥80 cm [27]; in addition, a higher frequency of plasma calcidiol deficiency was observed in women with WC ≥ 80 cm (85.4%) than those with WC < 80 cm (14.6%). It supports the evidence of low circulating vitamin D levels since it is stored in adipose tissue [47,48]; in obesity, larger amounts of adipose tissues sequester or buffer calcidiol, contributing to its low status [49,50]. In addition, the use of some antilipemic drugs, such as statins, may affect serum calcidiol concentrations [51], and some studies suggested an increase [52] or no effect on calcidiol levels [53].

Median blood pressure in both groups was reported as normal, although in the MetS group some women received antihypertensive treatment; therefore, they had normal SPB and DBP levels. In the current study, no association was shown between vitamin D status and hypertension (*p* = 0.054). A similarity was found in adult women with obesity in Paraguay that found no association between calcidiol deficiency and hypertension [54]. However, another study of postmenopausal women in Colombia reported that serum calcidiol deficiency was a risk factor for hypertension (OR 1.47, 95% CI: 1.36–1.58) [13]. These results showed that among women with calcidiol deficiency, higher SBP and DBP were significantly associated with the presence of MetS. According to the current OR Model 2, each unit increase in SBP or DBP was associated with a 6.7–7.5% increase in the odds of having MetS (OR: 1.067–1.075, *p* < 0.05). Similar associations were observed when vitamin D insufficiency and deficiency were combined (<30 ng/mL), with each unit increase in blood pressure associated with a 6.1–7.0% increase in the odds of MetS (OR: 1.061–1.070, *p* < 0.05). These associations may be due to the fully active vitamin D form, calcitriol, which inhibits the synthesis of the hormone renin [22], leading to a decrease in blood pressure [55]; however, at deficient calcidiol levels, there is increased renin activity, vascular dysfunction, and development of hypertension [9,56].

In the current study, hypertriglyceridemia (≥150 mg/dL) was not significantly associated with calcidiol levels. However, a previous study of Peru women over 20 years of age reported that calcidiol deficiency increases the risk of elevated triglycerides up to fourfold (OR: 4.07, 95% CI: 1.8–9.3) [57]. This could be due to the relationship of serum calcidiol deficiency and lipid metabolism, leading to increased triglyceride concentration, accompanied by decreased HDL-cholesterol levels [58]. Every 1-unit increase in triglycerides was associated with a 1.7–1.8% increase in the odds of metabolic syndrome, at both calcidiol status of deficiency and below 30 ng/mL, based on the two proposed regression models (OR: 1.017–1.018, *p* < 0.001). The presence of MetS was also associated with HDL-cholesterol in women with deficient vitamin D status: for each unit increase in HDL-cholesterol, the likelihood of MetS decreased by 6.0–8.2% (OR: 0.918–0.940, *p* < 0.05), while at concentrations above 30 ng/mL, significance was determined in the second model which adjusted for the use of antilipemic treatment; each unit increase in HDL-cholesterol was associated with a 6.1% reduction in the odds of having MetS (OR: 0.939, 95% CI: 0.886–0.996, *p* = 0.036). This finding shows that the protective effect of HDL-cholesterol on metabolic health remains evident even when the influence of pharmacological treatment is considered. In addition, these results are of clinical relevance since postmenopausal women tend to present higher risk of having low HDL-cholesterol levels [59,60]. According to the Endocrine Society, the International Osteoporosis Foundation (IOF) and the European Society for Clinical and Economic Aspects of Osteoporosis and Osteoarthritis (ESCEO) [6,7], vitamin D levels at or above 30 ng/mL (75 nmol/L) has been stated as protective at this stage of aging, and clinical practice suggests improved diet and supplementation intervention of vitamin D.

The involvement of vitamin D, as calcitriol, in the regulation of glucose by stimulating insulin secretion and improving sensitivity [24,25,61] may reflect the findings from a study in Cuban women aged 50 to 59 years, in which an association was reported between high serum glucose levels (>100 mg/dL) and deficient serum vitamin D levels (*p* = 0.013) [24]. In the current study, it was reported that women with normal serum glucose levels (<100 mg/dL) had a higher proportion of sufficient calcidiol levels (67.6%) compared to those with hyperglycemia (32.4%); however, no association was determined (*p* = 0.309). Based in both regression models, at deficient and below 30 ng/mL calcidiol levels, it is suggested that each increased unit in glucose concentration was associated with an 8.3–9.1% increase in the odds of having MetS (OR: 1.083–1.091, *p* < 0.05). The consistency of this association across both regression models shows that the relationship remained significant even after adjustment for potential confounding variables.

MetS, characterized by insulin resistance, obesity, and dyslipidemia, disrupts normal phosphate homeostasis, often leading to a chronic, low-grade elevation in serum phosphate or hyperphosphatemia, and impairing glucose metabolism and significantly increasing cardiovascular and cardiometabolic risks [62,63,64,65]. Phosphate balance is tightly controlled by a complex endocrine network: insulin resistance and systemic inflammation induce target-organ resistance to FGF23 and PTH, impairing the kidney’s ability to excrete excess phosphate [62,64]. Fibroblast Growth Factor 23 (FGF23) is secreted by osteocytes; it is the primary phosphaturic hormone, which promotes renal phosphate excretion, suppresses renal 1α hydroxylase, and reduces the synthesis of active vitamin D production, which may be a mechanism to reduce intestinal phosphate absorption [66,67].

Then, there is an inverse relationship between MetS and vitamin D, because vitamin D enhances intestinal phosphate absorption, but it is suppressed by high FGF-13 levels [68,69]. Parathyroid Hormone (PTH) promotes renal phosphate excretion by reducing the expression of sodium-phosphate cotransporters (NaPi-IIa and NaPi-IIc) in the proximal tubule [66,68]. The Klotho, an essential co-receptor, enables FGF-23 to bind to its target receptors in the kidneys [68,69,70,71,72]. Therefore, there is a breakdown of the physiology of phosphate metabolism in the context of metabolic syndrome and hyperphosphatemia. The rise in vitamin D levels is beneficial for MetS.

## 5. Strengths and Limitations

The main strengths of the current study are to demonstrate the high prevalence of MetS and calcidiol (25D_3_) deficiency in Mexican women aged 40–60 years, as well as the association between MetS components and calcidiol blood levels. Another one of its strengths was the collection of several complementary methodologies. The main limitation of the current study is the cross-sectional design that avoids establishing causal interferences on whether calcidiol deficiency contributed to the development of MetS; therefore, prospective analysis should be developed in future research. Because of the convenience sample, the study may have limited generalizability to 40–60-year-old women living in the northeast of Mexico. In addition, the sample size was limited to 202 women, which may reduce statistical power. Finally, the lack of control of some variables like estrogen or other hormone concentrations, participants’ skin pigmentation, duration of sun exposure, and multiple confounders (such as dietary habits, sedentary or indoor lifestyle, or MetS medication) which were not addressed in this study can influence calcidiol status and metabolic health.

## 6. Conclusions

This study demonstrated a high prevalence of metabolic syndrome (59.9%) and calcidiol deficiency (67.8%) among women aged 40–60 years in the northeast of Mexico. Most participants had deficient or insufficient calcidiol levels, while dietary intake of vitamin D was inadequate in both NoMets and MetS groups—up to half and one third of that recommended for Mexican women. Besides low vitamin D intakes, low calcidiol levels may be due to impaired hepatic function due to chronic inflammatory mechanisms which affect its conversion to calcidiol via hepatic 25-hydroxylase CYP2R1.

Although calcidiol status was not independently associated with individual MetS components, regression analyses showed significant associations between the presence of MetS and blood pressure, triglycerides, HDL-cholesterol, and glucose concentrations at deficient and suboptimal calcidiol levels. These results suggest that vitamin D may play a role in cardiometabolic health through its involvement in lipid metabolism, glucose regulation, insulin sensitivity, and vascular function. Reduced vitamin D levels in the context of MetS may reflect adaptive suppression driven by phosphate overload rather than true deficiency. However, vitamin D targets or supplementation as a protective strategy against MetS cannot be recommended, as the study design and the underlying phosphate physiology do not support such clinical implications. Maintaining calcidiol concentrations at or above 30 ng/mL may therefore represent a potential strategy to support metabolic health in midlife and postmenopausal women, although longitudinal and intervention studies are needed to clarify causality and evaluate the benefits of vitamin D on health.

## Figures and Tables

**Table 1:** Metabolic syndrome components, calcidiol (25D_3_) status and vitamin D intake in 40–60-yr-old women.

	NoMetS (*n* = 81)	MetS (*n* = 121)	*p*
Age (years)	50.0 (45.5–53.0)	51.0 (47.0–55.0)	0.129
BMI (kg/m^2^)	25.8 (23.2–29.4)	30.8 (27.7–34.8)	<0.001 **
Metabolic Syndrome Components			
WC (cm)	85.5 (76.0–92.0)	97.0 (91.2–105.8)	<0.001 **
Blood pressure			
SBP (mmHg)	109.0 (101.5–115.0)	123.0 (115.0–135.5)	<0.001 **
DBP (mmHg)	68.0 (63.0–73.0)	79.0 (69.0–86.0)	<0.001 **
Biochemical parameters			
Triglycerides (mg/dL)	105.0 (91.5–128.0)	164.0 (132.0–217.0)	<0.001 **
HDL-cholesterol (mg/dL)	35.0 (29.1–47.9)	32.0 (27.5–37.7)	0.006 *
Glucose (mg/dL)	86.0 (82.0–95.0)	98.0 (89.0–109.0)	<0.001 **
Calcidiol (25D3) status (ng/mL)	17.2 (5.9–29.3)	9.0 (3.3–20.4)	0.003 *
Deficient (<20 ng/mL)	46 (56.8%)	91 (75.2%)	<0.001 **
Insufficient (21–29 ng/mL)	15 (18.5%)	13 (10.7%)	0.705
Sufficient (>30 ng/mL)	20 (24.7%)	17 (14.0%)	0.622
Vitamin D intake			
Diet (IU/day)	194.0 (97.3–336.6)	120.3 (81.5–231.5)	0.010 *
Season of blood withdrawn			
Spring	13 (16.0%)	23 (19.0%)	0.852
Summer	48 (59.3%)	72 (59.5%)	0.945
Autum	17 (21.0%)	14 (11.6%)	0.060
Winter	3 (3.7%)	12 (9.9%)	0.058

BMI: body mass index, WC: waist circumference, SBP: systolic blood pressure, DBP: diastolic blood pressure, IU/day: international units per day. Numerical data is shown as median (quartiles) (Q1–Q3). Differences between groups by Mann–Whitney-U test, *p* < 0.05 *, *p* < 0.001 **. Categorical data is shown as *n* (%), frequency (percentage). Differences between groups by Chi-squared test, *p* < 0.05 *, *p* < 0.001 **.

**Table 2:** MetS components and groups of calcidiol (25D_3_) status.

	Calcidiol (25D_3_) Status				
MetS Components	Deficiency <20 ng/mL (*n* = 137)	Insufficiency 21–29 ng/mL (*n* = 28)	Sufficiency >30 ng/mL (*n* = 37)	Total (*n* = 202)	*p*
WC					
Normal	20 (14.6)	9 (32.1)	8 (21.6)	37 (18.3)	0.078
≥80 cm	117 (85.4)	19 (67.9)	29 (78.4)	165 (81.7)	
BP					
Normal	86 (62.8)	19 (67.9)	31 (83.8)	136 (67.3)	0.054
≥130/85 mmHg or antihypertensive treatment	51 (37.2)	9 (32.1)	6 (16.2)	66 (32.7)	
Triglycerides					
Normal	79 (57.7)	16 (57.1)	24 (64.9)	119 (58.9)	0.717
≥150 mg/dL or antilipemic treatment	58 (42.3)	12 (42.9)	13 (35.1)	83 (41.1)	
HDL-cholesterol					
Normal	12 (8.8)	2 (7.1)	3 (8.1)	17 (8.4)	0.959
<50 mg/dL or specific treatment	125 (91.2)	26 (92.9)	34 (91.9)	185 (91.6)	
Glucose					
Normal	93 (67.9)	23 (82.1)	25 (67.6)	141 (69.8)	0.309
≥100 mg/dL or antidiabetic treatment	44 (32.1)	5 (17.9)	12 (32.4)	61 (30.2)	
Presence of MetS					
No	46 (33.6)	15 (53.6)	20 (54.1)	81 (40.1)	0.023 *
Yes	91 (66.4)	13 (46.4)	17 (45.9)	121 (59.9)	

Data is shown as *n* (%), frequency (percentage). WC: waist circumference, BP: blood pressure, MetS: metabolic syndrome. Differences among groups by Chi-squared test, *p* < 0.05 *.

**Table 3:** Association of the presence of MetS with its components according to groups of calcidiol (25D_3_) levels in 40-to-60-year-old women.

MetS Components	Model 1 ^a^			Model 2 ^b^		
	OR	95% CI	*p*	OR	95% CI	*p*
Deficiency ≤ 20 ng/mL (*n* = 91)						
WC (cm)	1.057	0.967–1.154	0.221	1.055	0.955–1.165	0.293
SBP (mmHg)	1.068	1.010–1.128	0.020 *	1.067	1.006–1.130	0.029 *
DBP (mmHg)	1.060	0.999–1.126	0.056	1.075	1.007–1.147	0.030 *
Triglycerides (mg/dL)	1.017	1.008–1.026	<0.001 **	1.018	1.008–1.027	<0.001 **
HDL-cholesterol (mg/dL)	0.939	0.886–0.995	0.033 *	0.918	0.856–0.984	0.016 *
Glucose (mg/dL)	1.083	1.026–1.144	0.004 *	1.089	1.023–1.158	0.007 *
Below 30 ng/mL (Deficiency ≤ 20 ng/mL + Insufficiency 21–29 ng/mL) (*n* = 104)						
WC (cm)	1.090	1.006–1.182	0.035 *	1.089	0.999–1.187	0.054
SBP (mmHg)	1.062	1.008–1.118	0.023 *	1.061	1.005–1.120	0.031 *
DBP (mmHg)	1.056	0.999–1.116	0.054	1.070	1.007–1.136	0.028 *
Triglycerides (mg/dL)	1.018	1.009–1.027	<0.001 **	1.018	1.009–1.028	<0.001 **
HDL-cholesterol (mg/dL)	0.951	0.902–1.003	0.062	0.939	0.886–0.996	0.036 *
Glucose (mg/dL)	1.084	1.031–1.139	0.002 *	1.091	1.032–1.153	0.002 *
Sufficiency ≥ 30 ng/mL (*n* = 17)						
WC (cm)	17.401	0.000	0.998	1.966	0.000	1.000
SBP (mmHg)	7.855	0.000	0.997	4.376	0.000	0.998
DBP (mmHg)	15.992	0.000	0.997	4.638	0.000	0.999
Triglycerides (mg/dL)	1.384	0.000–3.073	0.997	1.516	0.000–9.377	0.998
HDL-cholesterol (mg/dL)	0.255	0.000	0.998	0.153	0.000	0.998
Glucose (mg/dL)	2.376	0.000	0.998	3.441	0.000	0.998

WC: waist circumference, SBP: systolic blood pressure, DBP: diastolic blood pressure, MetS: metabolic syndrome. Data analyzed by logistic regression analysis, OR: odds ratio, 95% CI: 95% confidence interval, significance *p* < 0.05 *, *p* < 0.001 **. ^a^ Adjusted for age, stage of aging, and body mass index, ^b^ Adjusted for age, stage of aging, body mass index, vitamin D intake, season at blood sample extraction, use of antilipemic and hormonal treatment.

## Data Availability

The data presented in this study are available on request from the corresponding author due to the signed consent agreements around data sharing, which only allow access to external researchers for studies following the project purposes.
